# Beyond efficiency: reimagining health technology assessment in the age of artificial intelligence

**DOI:** 10.1017/S0266462326103778

**Published:** 2026-04-16

**Authors:** Manuel C. Cossio, Ramiro Gilardino

**Affiliations:** 1 Cytel Inc, USA; 2 Faculty of Medicine and Health Sciences, University of Barcelona, Spain; 3 Public Health Department, School of Medicine, University of Buenos Aires, Argentina

**Keywords:** artificial intelligence, evidence synthesis, health technology assessment, health efficiency, public policy

The increasing attention to artificial intelligence (AI), particularly generative AI and agentic AI, within the health technology assessment (HTA) community reflects a broader recognition that digital technologies are reshaping the generation, synthesis, and use of evidence in decision making. The recent Global Policy Forum (GPF) convened by Health Technology Assessment International (HTAi) provides a timely and thoughtful overview of the opportunities and challenges associated with integrating AI into HTA activities. The discussion highlights several critical themes, including trust in AI systems, the importance of maintaining human agency in decision making, and the need for risk-based approaches to implementation (1). These themes provide an important foundation for the responsible adoption of AI in HTA.

Simultaneously, much of the current discussion understandably focuses on how AI can improve the efficiency of specific HTA tasks, such as systematic literature reviews, database analysis, data extraction, evidence synthesis, and economic modeling (2). These operational improvements are significant. HTA processes often involve labor-intensive and time-consuming evidence generation activities, and AI-enabled tools can support the faster and potentially more consistent synthesis of growing bodies of clinical and real-world evidence.

While much current discussion focuses on AI’s efficiency gains in HTA, this article argues for AI’s transformative potential to extend beyond task automation. We propose that AI can enable a broader evolution in HTA systems, shifting from episodic, static assessments to more adaptive, continuously learning decision-support systems, thereby serving as a catalyst for rethinking HTA’s fundamental structure and function.

## AI as an enabler of life cycle and dynamic HTA

Traditional HTA processes were designed in an era when evidence generation followed a linear trajectory. Clinical trials produce data that are synthesized into assessments, which then inform reimbursement or coverage decisions. While reassessments sometimes occurred, they were infrequent and triggered by major evidence updates or policy changes (3;4).

In contrast, today’s health innovation ecosystem continuously generates evidence across the life cycle of technologies. The volume and complexity of available information are expanding due to the use of real-world data, digital health tools, and post-market evidence generation. Consequently, HTA systems increasingly face the challenge of maintaining relevance in an environment where evidence evolves more rapidly than traditional assessment cycles allow (3). AI may play a critical role in enabling new models of life cycle or living HTA, where evidence synthesis and decision support occur continuously rather than at discrete intervals. AI tools can assist in continuously monitoring emerging literature, identifying relevant new studies, updating evidence syntheses, and highlighting signals that may warrant reassessment of existing decisions (5;6). Such capabilities could support more adaptive and responsive HTA processes that better reflect the evolving nature of clinical and economic evidence.

Importantly, these developments should not be interpreted as a replacement for human judgment. Rather, they suggest the emergence of what might be described as augmented HTA, in which AI enhances the ability of experts to process large volumes of information while preserving the essential role of human interpretation, contextualization, and value-based decision making.

Therefore, the emphasis on human agency highlighted in GPF discussions is well placed. AI systems can assist in identifying patterns, summarizing evidence, and generating analytical results. However, HTA decisions ultimately require normative judgments about value, uncertainty, equity, and societal priorities. Maintaining human oversight ensures that these value-laden decisions are anchored in transparent and accountable governance structures.

## Moving from automation to methodological integration

Although the potential applications of AI in HTA are rapidly expanding, the methodological foundations for its responsible use remain under development. GPF discussions emphasize the importance of validation, transparency, and trust-building in AI-enabled HTA processes (1). These concerns highlight the need for methodological standards that clarify how AI tools should be evaluated, validated, and reported when used in HTA-related activities.

Several questions arise in this context. What constitutes acceptable performance when AI tools assist in tasks such as literature screening or data extraction? How should the outputs of AI-assisted analyses be documented and audited? To what extent should HTA submissions disclose the use of AI tools in preparing evidence dossiers? How can reproducibility be ensured when AI systems may generate different outputs depending on the prompts, model versions, or training data?

Addressing these questions requires the development of shared methodological frameworks. One useful starting point may be to distinguish between the different levels of AI involvement within HTA workflows.

At the most basic level, AI can perform *assistive functions*, such as generating search strings, summarizing text, translating documents, or formatting outputs. These applications involve relatively low levels of analytical risk and can often be validated using straightforward quality checks.

The second category involves *analytical support*, including literature screening, data extraction, statistical analysis, and support for economic modeling. These activities may influence the structure of evidence syntheses and, therefore, require more robust validation procedures and transparency regarding the methods and model performance.

The third and more complex level involves *decision-support applications*, where AI tools assist in synthesizing evidence across domains or identifying potential policy implications. While such tools may eventually become valuable in supporting deliberative processes, their use raises additional governance and accountability issues.

Developing clear guidance on these distinctions could help HTA organizations and stakeholders implement risk-proportionate oversight mechanisms consistent with broader regulatory approaches to AI governance.

## AI across the HTA evidence ecosystem

Another dimension of AI integration that deserves greater attention is its role in supporting the broader HTA evidence ecosystem. Much of the current focus is on upstream evidence generation activities; however, AI may also play an important role in improving how HTA outputs are communicated, interpreted, and used by stakeholders.

HTA decisions affect a wide range of stakeholders, including patients, clinicians, policy makers, and the public. However, the technical nature of HTA reports often limits their accessibility to specialized audiences. Therefore, enhancing the transparency and accessibility of HTA outputs is an important component of strengthening trust in assessment processes.

Generative AI tools may offer new opportunities to support *evidence translation*, including the development of plain language summaries and other accessible formats that communicate HTA findings to broader audiences (7). Though assisting in the drafting and adaptation of complex technical content into understandable narratives, AI can help bridge the gap between technical evidence synthesis and stakeholder engagement.

However, as with other AI applications, the use of AI for evidence translation requires careful oversight. Ensuring that AI-generated summaries accurately reflect the underlying evidence and avoid oversimplification or bias will remain a critical responsibility of HTA experts and communication specialists. When appropriately governed, AI-assisted PLS development can contribute to greater transparency and inclusivity in HTA processes.

## Governance and institutional adaptation

The integration of AI into HTA workflows will also require institutional-level adaptation. HTA agencies and organizations must develop governance structures capable of overseeing AI-enabled processes to ensure methodological rigor and transparency.

This may involve several complementary actions. First, HTA agencies may need to establish policies regarding the acceptable use of AI tools in HTA submissions, including disclosure requirements for AI-assisted analysis. Such policies would help ensure that reviewers and decision makers understand how the evidence was generated and synthesized (8).

Second, HTA agencies may need to invest in their internal capacity to evaluate AI-generated outputs. As AI tools become more prevalent in evidence synthesis and modeling, HTA reviewers will need the skills and resources necessary to assess the validity and limitations of AI-assisted analysis.

Third, opportunities may arise to develop shared infrastructures or collaborative platforms that support the validation and benchmarking of AI tools used in HTA activities. Such initiatives could help avoid fragmentation and duplication of efforts across jurisdictions while promoting transparency and methodological consistency.

Finally, governance frameworks should consider the ethical implications of AI use, including issues related to data privacy, intellectual property, algorithmic bias, and accountability. These considerations align with broader international efforts to establish responsible AI governance in health systems.

## Global equity and capacity considerations

The global HTA community includes countries with widely varying institutional capacities and methodological resources. In this context, the adoption of AI tools could have divergent implications for global equity issues.

On the one hand, AI technologies may lower barriers to HTA implementation by automating resource-intensive evidence-synthesis tasks. For emerging HTA systems and low- and middle-income countries (LMICs), access to AI-supported tools could accelerate the development of local assessment capacity and support evidence-informed decision making.

However, if access to advanced AI tools remains concentrated within well-resourced organizations or proprietary platforms, disparities in HTA capacity could widen. Therefore, ensuring that AI integration contributes to greater global equity will require deliberate efforts to promote open standards, shared resources, and collaborative knowledge exchange.

Initiatives such as communities of practice, as proposed in GPF discussions, could play an important role in fostering collaboration and sharing experiences with AI-enabled HTA methodologies. International organizations, academic institutions, and HTA networks may also contribute by supporting training, capacity building, and developing open methodological guidance.

## Toward an augmented HTA paradigm

Discussions emerging from the GPF highlight a growing consensus that the integration of AI into HTA should proceed cautiously, with strong attention to transparency, validation, and ethical governance. These principles provide an essential foundation for responsible innovations.

Simultaneously, the HTA community has an opportunity to consider more fundamentally how AI might support the evolution of HTA systems. Rather than viewing AI solely as a means of accelerating existing processes, it may be more productive to consider how AI can contribute to a more adaptive, transparent, and inclusive HTA ecosystem.

Such a paradigm would combine the analytical capabilities of AI with the interpretive and normative expertise of human decision makers. AI can assist in continuously monitoring emerging evidence, support dynamic evidence synthesis, and enhance communication with stakeholders. Human experts would remain responsible for interpreting evidence, weighing societal values, and making accountable policy decisions.

In this *augmented HTA paradigm*, AI becomes a powerful tool that supports, rather than replaces, the deliberative processes at the heart of HTA. Combining technological innovation with strong governance and methodological rigor, the HTA community can harness the benefits of AI while preserving the principles that underpin evidence-based health policy.

Ultimately, the successful integration of AI into HTA will depend not only on technological advances but also on the willingness of institutions and stakeholders to collaborate, experiment responsibly, and continuously refine the frameworks that guide decision making in an increasingly complex evidence landscape.
